# Severity of Retrognathia and Glossoptosis Does Not Predict Respiratory and Feeding Disorders in Pierre Robin Sequence

**DOI:** 10.3389/fped.2018.00351

**Published:** 2018-11-20

**Authors:** Anne Morice, Véronique Soupre, Delphine Mitanchez, Francis Renault, Brigitte Fauroux, Sandrine Marlin, Nicolas Leboulanger, Natacha Kadlub, Marie-Paule Vazquez, Arnaud Picard, Véronique Abadie

**Affiliations:** ^1^Department of Maxillofacial and Plastic Surgery, APHP, Hôpital Universitaire Necker-Enfants Malades, Université Paris Descartes, Sorbonne Paris Cité, Paris, France; ^2^Rare Diseases Reference Center Coordinator for Clefts and Facial Malformations, APHP, Hôpital Universitaire Necker-Enfants Malades, Paris, France; ^3^Department of Perinatality, APHP, GHUEP, Armand Trousseau Hospital, Medicine Sorbonne University, Paris, France; ^4^Pediatric Neurophysiology Unit, APHP, Armand-Trousseau University Hospital, Paris, France; ^5^Pediatric Noninvasive Ventilation and Sleep Unit, APHP, Hôpital Necker Enfants-Malades, Paris Descartes University, Paris, France; ^6^Department of Genetics, APHP, Hôpital Universitaire Necker-Enfants Malades, Université Paris Descartes, Sorbonne Paris Cité, Paris, France; ^7^Department of Otorhinolaryngology and Head and Neck Surgery, APHP, Hôpital Universitaire Necker-Enfants Malades, Université Paris Descartes, Sorbonne Paris Cité, Paris, France; ^8^Department of Pediatrics, APHP, Hôpital Universitaire Necker-Enfants Malades, Université Paris Descartes, Sorbonne Paris Cité, Paris, France; ^9^National Reference Center for Pierre Robin Sequence, APHP, Hôpital Universitaire Necker-Enfants Malades, Paris, France

**Keywords:** Pierre Robin sequence, retrognathia, glossoptosis, respiratory disorders, feeding disorders

## Abstract

Pierre Robin sequence (PRS) may lead to life-threatening respiratory and feeding disorders. With the aim to analyse the association of the severities of retrognathia and glossoptosis with those of respiratory and feeding disorders, we retrospectively studied a series of 50 infants with retrognathia, glossoptosis, cleft palate, and airway obstruction. The patients were managed from birth to at least 6 years of age by a single pediatric team at the Armand Trousseau Hospital in Paris within a 12 years period (2000–2012). Retrognathia and glossoptosis were graded in the neonatal period according to a specific clinical examination. Ventilation assistance was required for 32/50 (64%) patients, and enteral feeding for 41/50 (82%). The grades of retrognathia and glossoptosis and the severity of respiratory disorders did not differ between patients with isolated PRS and syndromic PRS. Severe respiratory disorders were more common and long-lasting feeding (>12 months) was more frequently required in patients with syndromic PRS compared with isolated PRS (42 vs. 13%, *p* = 0.04 and 42 vs. 4%, *p* < 0.01 respectively). Using univariate analysis, neurological impairments and laryngomalacia were associated with severe respiratory disorders [Odds ratio (OR) 5.0, 95% CI 1.3–19.6; and OR 14.6, 95% CI 1.3–161.4; *p* < 0.05] as well as with long-lasting feeding (>12 months) disorders (OR 18.6, 95% CI 3.9–89.2 and OR 20.4, 95% CI 3,4–122.8; *p* < 10^−2^). Syndromic SPR status was also associated with severe respiratory disorders (OR 4.9, 95% CI 1–32.5; *p* < 0.05). Using multivariate analysis, only syndromic PRS status was predictive for severe respiratory disorders (adjusted OR 8, 95% CI 1.47–44.57; *p* < 0.05); and only neurological impairments remained a significant risk for long lasting feeding disorders (>12 months) (adjusted OR 21.72, 95% CI 3.4–138.63; *p* < 10^−2^). The grades of retrognathia and glossoptosis were not predictive factors for the severity of respiratory and feeding disorders.

**Conclusion**: In children with PRS, the severity of clinical conditions may not correlate with anatomic variables but rather with laryngeal abnormalities, neurological impairement and syndromic PRS status.

## Introduction

Pierre Robin sequence (PRS), originally described as associating retrognathia, glossoptosis and neonatal upper airway obstruction, has been secondarily considered a malformation sequence arising from limited mandibular growth and a vertical tongue position, leading to a U-shaped cleft palate (1, 2). PRS can be isolated or syndromic, depending on associated signs and genetic assessment (3–6). Although mortality rate for children with PRS has significantly decreased, it remains substantial, up to 16.6% according to latest reports (7, 8).

PRS is heterogeneous in terms of both diagnostic criteria and clinical consequences. Predicting the severity of respiratory and digestive disorders in newborn infants with PRS could help to reduce both morbidity and mortality rates and to choose the most suitable treatment. Due to the lack of consensual guidelines for assessing and managing respiratory and feeding disorders in the neonatal period, therapeutic options widely vary among pediatric teams and comparing the results of previous series is a difficult issue. Regarding airway obstruction, conservative treatments include prone position, palate plates, nasopharyngeal tube, and non-invasive ventilation; and surgical treatments include mandibular distraction osteogenesis (MDO), tongue-lip adhesion (TLA), and tracheostomy (9). Feeding disorders, mainly due to sucking–swallowing and esophageal dysfunctions (10), may cause failure to thrive and aspiration pneumonia and require enteral feeding using nasogastric tube (NGT) or gastrostomy, in up to 75% of patients with PRS (5, 11). Even if a direct causal link between anatomic features and functional disorders may be reasonably suspected, this has not been yet demonstrated.

Vipulananthan et al. (12) have reported that male gender and primary presentation with respiratory failure predicted more severe airway obstruction and the need for tracheostomy in patients with PRS. In their study, patients with PRS with cleft palate more frequently showed early respiratory failure than patients with no cleft. In our series of children with PRS and cleft palate, we aimed to report anatomic characteristics (retrognathia and glossoptosis), and clinical conditions (respiratory and feeding disorders), and analyze any association between these anatomic and functional variables, taking into account potential confounding factors such as additional airway anomalies and neurological impairment.

## Materials and methods

### Patients

We reviewed the database of the Armand Trousseau university hospital over a 12 years period (2000–2012) to identify patients with PRS who were managed by our pediatric multidisciplinary team from birth up to at least 6 years of age. We selected patients presenting with the association of retrognathia, glossoptosis, cleft palate, and airway obstruction. Airway obstruction was defined by the need for at least prone positioning. As a result of a multidisciplinary evaluation including clinical genetics, we classified PRS as isolated (iPRS) or syndromic (sPRS). Patients with associated clinical features or genetic anomalies were classified as sPRS even if the cause of the syndrome was not identified (i.e., syndromic means “non-isolated”).

### Anatomic variables

**Figure 1:**
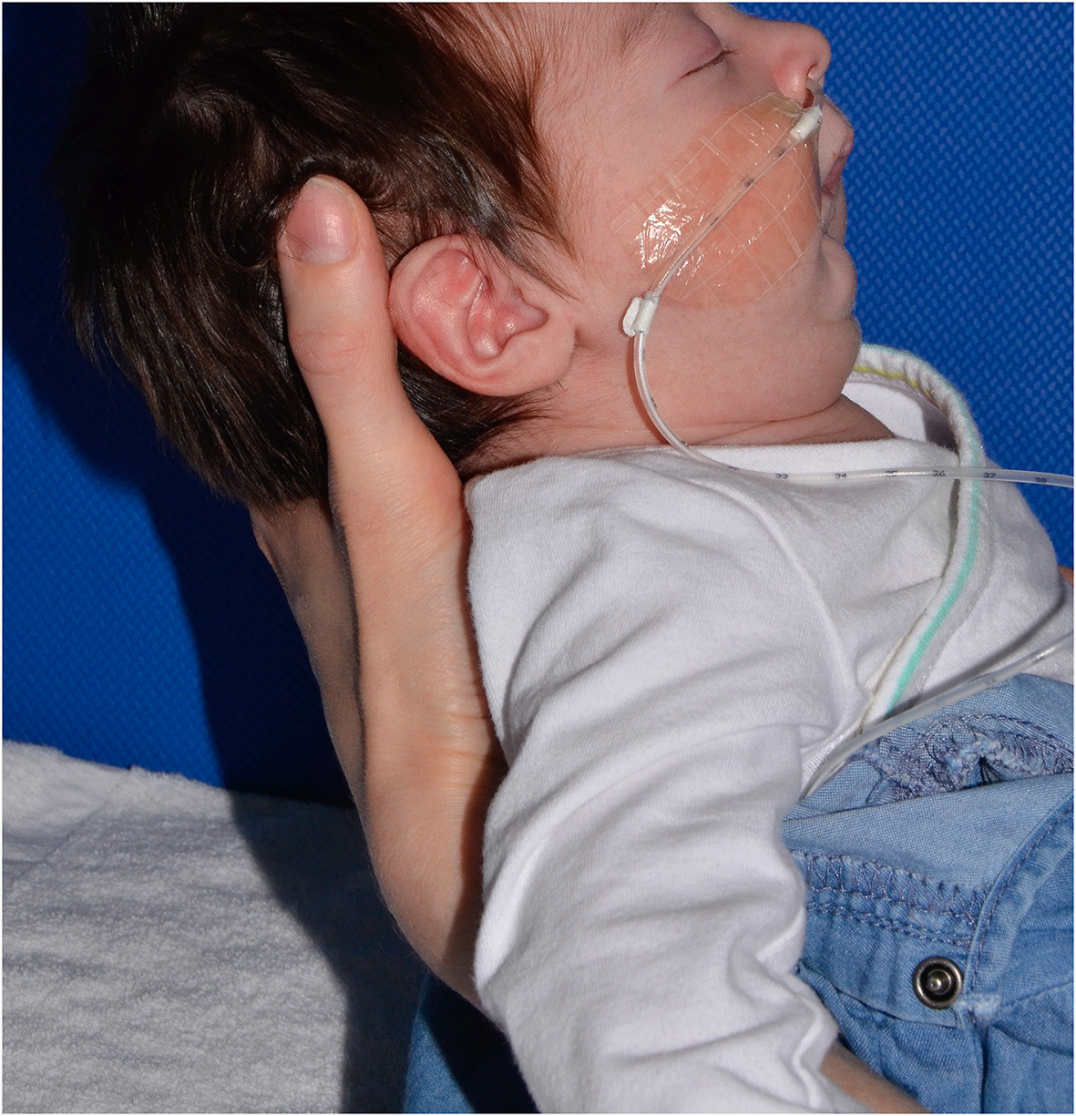
Lateral view of an infant with Pierre Robin sequence: examination in half-sitting position.

**Figure 2:**
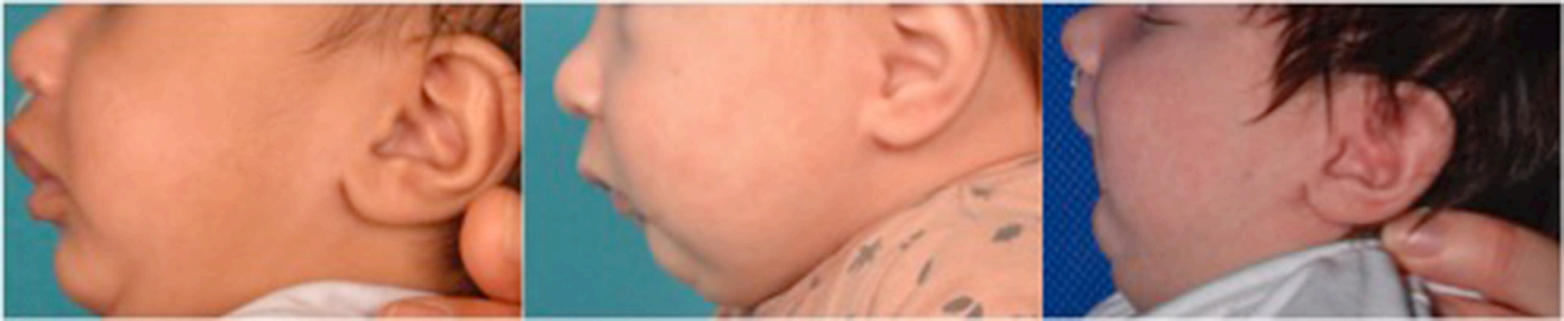
Grading system for retrognathia on lateral views of infants with Pierre Robin sequence (left: mild; middle: moderate; right: severe).

Retrognathia and glossoptosis were graded according to a specific clinical examination performed shortly after birth in a child in half-sitting position, awake, and calm (Figure 1, with parents' consent form). Retrognathia was graded as “severe” when the red lower lip was completely covered by the upper lip; moderate when it was partially apparent; and mild when it was completely apparent (Figure 2, with parents' consent form). Glossoptosis was graded as severe when the tongue was vertical and posteriorly placed, moderate in case of posterior ptosis but no vertical position of the tongue, and mild in case of sublingual crests rise.

### Respiratory disorders

We recorded the duration and type of ventilation assistance, including non-invasive continuous positive airway pressure (CPAP), tracheal tube, and tracheostomy. The results of airway endoscopy were analyzed to detect additional factors of airway obstruction including laryngomalacia, tracheomalacia, and any other anomalies. Based on both clinical and endoscopic examination, laryngomalacia was defined as severe in case of complete inspiratory laryngeal collapse, and as mild otherwise.

The indication and evaluation of the efficacy of ventilation treatments were based on systematic clinical assessment, oxymetry, and polygraphic data. Newborn patients who showed immediate desaturation (< 90%) and hypercapnia (pCO_2_ > 50 mmHg) received continuous CPAP. Newborn patients with Apnea Hypopnea Index (AHI) > 10/h and/or Oxygen Desaturation Index (ODI) > 15/h and/or minimal pulse oxymetry (SpO_2_ < 90% during more than 5% of polygraphic recording duration and/or maximal transcutaneous PtcCO_2_ > 50 mmHg) were treated using non-invasive CPAP during sleep only. Patients with normal or near-normal, oxymetry (SpO2 > 90%), were treated by prone positioning only. Respiratory disorders were graded as follows: mild with success of prone position only, moderate with success of non-invasive CPAP during sleep, and severe in case of tracheal intubation, failure of CPAP, or tracheostomy. In addition, we recorded the duration of invasive and non-invasive ventilation assistance.

### Feeding disorders

They were classified according to the duration of enteral feeding by NGT or gastrostomy. Enteral feeding was stopped when total oral diet was achieved with no aspiration episodes and normal weight growth. Disorders were considered very mild when enteral feeding was stopped within the first 3 weeks of life, mild between 3 weeks and 3 months of life, moderate between 3 and 6 months, severe between 6 and 12 months, and very severe > 12 months.

### Statistical analysis

Quantitative variables are described with median and interquartile range [25–75th percentile]. Categorical variables are described with frequencies. Categorical data were analyzed by chi-square test or Fisher exact test. Student t test or Mann-Whitney test were used to compare two groups and the Kruskal Wallis test with Dunn post-test to compare more than two.

Multivariate analyses were then conducted through logistic regression models involving respiratory disorders for one model, feeding disorders in a second model as binary dependent variables. The year of birth, prematurity and gender were included as confounding factors; PRS status, retrognathia, glossoptosis, laryngomalacia, and neurological impairments were included as independent potential predictor factors. The step-by-step variable selection procedure was used to keep only most parsimonious models (stepwise with the Akaike's information criteria). The Hosmer- Lemeshow's goodness of fit test was used for each model.

Results of univariate and multivariate analysis were reported as Odds Ratio (OR) and adjusted OR (aOR), respectively.

All test were two-sided. *P* < 0.05 was considered statistically significant. GraphPad Prism 5 and R (version 3.4.3-cran.r-project.org) were used for statistical analysis.

## Results

### Patients

Over the study period, we identified 60 infants with PRS who were managed and followed from birth to at least 6 years of age. We excluded 10 patients who received treatment initially or secondarily in another center. Thus, we included 50 patients (26 females; F/M ratio: 1.08). PRS was isolated in 24 patients (48%) and syndromic in 26 (52%) (Table 1). Prematurity rate was 8% (*n* = 4).

**Table 1:** Type and repartition of PRS (*n* = 50).

**Isolated PRS**	**24 (48%)**
Syndromic PRS	26 (52%)
Recognizable syndrome^*^	8
Chromosomal anomalies	3
Undefined syndrome	15

* *including Charge syndrome (n = 2), Williams Beuren, Stickler, Bamforth, Treacher, and cerebrocostomandibular syndrome (n = 1 each)*.

Retrognathia was mild in seven patients (14%), moderate in 32 (64%), and severe in 11 (22%). Glossoptosis was mild in 11 patients (22%), moderate in 23 (46%), and severe in 16 (32%). These frequencies did not significantly differ when comparing patients with iPRS and sPRS.

Neurological impairments were observed in 13 patients. Unexpected sudden death occurred in 2 patients, a 1-year-old child with chronic respiratory failure, and a 6-year-old child who acquired acute pneumonia without underlying respiratory disorders.

### Anatomic variables and respiratory disorders

Prone positioning alone was successful for 18/50 patients (36%) (Table 2). Additional ventilation assistance was needed in 32 patients (64%), including 20 who received non-invasive CPAP. A tracheal tube was used in 14 patients (28%) with acute respiratory distress. Tracheostomy was needed in five patients, including four with sPRS and one with iPRS, because of CPAP failure (*n* = 4) or severe acute respiratory distress (*n* = 1). Median duration of ventilation assistance was 24 days [1–138] (excluding patients with tracheostomy). Median duration of tracheal intubation before tracheostomy was 21 days [12–90]. Median time spent with tracheostomy was 21 months [13–36].

**Table 2:** Severe ventilation problems are more frequently observed in syndromic than in isolated Pierre Robin sequence.

**Ventilation problems**	**Total PRS (*n* = 50; %)**	**iPRS (*n* = 24; % of iPRS)**	**sPRS (*n* = 26; % of sPRS)**
Mild (prone position only)	22 (44%)	14 (58%)	8 (31%)
Moderate (non invasive CPAP)	14 (28%)	7 (29%)	7 (27%)
Severe (TT, tracheostomy)	14 (28%)	3 (13%)	11 (42%)^*^

*CPAP, continuous positive airway pressure; TT, tracheal tube; iPRS, isolated PRS; sPRS, syndromic PRS*.

* *p = 0.04 by Fisher test*.

Respiratory disorders were graded as mild, moderate, and severe in 44, 28, and 28% of the patients, respectively. Severe respiratory disorders were more frequently observed in patients with sPRS than in those with iPRS (*p* = 0.04; Table 2). The duration of invasive or non-invasive ventilation assistance tended to be longer in patients with sPRS (135 days [1-365]) than in patients with iPRS (45 days [4–100.5]).

Among the patients who required ventilation assistance, retrognathia was severe in 4, moderate in 20, and mild in 4; glossoptosis was severe in 10, moderate in 11, and mild in 7. Severity of respiratory disorders and duration of ventilation assistance showed no significant differences according to the grades of retrognathia and glossoptosis (Table 3).

**Table 3:** Median duration (days) of ventilation assistance (excluding patients treated by prone position only) by grades of retrognathia, glossoptosis and laryngomalacia.

	**Grades**		
	**Mild**	**Moderate**	**Severe**	***p*-value**
Retrognathia	142.5 [101.5–172.6] (*n* = 4)	15 [1–136.5] (*n* = 20)	287.5 [45–1095] (*n* = 9)	NS^*^
Glossoptosis	54 [36.2–202.5] (*n* = 7)	6 [1–35] (*n* = 12)	141 [63.7–309.9] (*n* = 14)	NS^*^
Laryngomalacia	150 [3–240.6] (*n* = 5)	/	1,825 [1003.7–2258.7] (*n* = 4)	*p* = 0.01^**^

Data are expressed as median and interquartile range [25–75th percentile]. NS, non-significant;

* by Kruskal-Wallis test,

** *by Mann-Whitney test*.

All patients who required ventilation assistance underwent airway endoscopy. Laryngomalacia was reported in 9 patients. It was classified as mild in five patients and severe in four. Ventilation assistance was more prolonged in patients with severe than in those with mild laryngomalacia (*p* = 0.01; Table 3).

Using univariate analysis, sSPR, neurological impairments and laryngomalacia were associated with severe respiratory disorders (OR 4.9, 95% CI 1–32.5; OR 5.0, 95% CI 1.3–19.6; and OR 14.6, 95% CI 1.3–161.4; *p* < 0.05).

Using multivariate analysis, the grades of retrognathia and glossoptosis, and laryngomalacia, were not predictive factors for severe respiratory disorders. Neurological impairments were not significant either. Only sPRS remained significant for predicting severe respiratory disorders (aOR 8, 95% CI 1.47–44.57; *p* < 0.05) in this model including also the year of birth, prematurity and gender. To note, these three confounding factors were not statistically significant.

### Anatomic variables and feeding disorders

Among the 50 patients, 41 (82%) required enteral feeding by NGT, including seven for whom gastrostomy was secondary performed because of long-duration NGT feeding or associated gastroenteral disorders. The median duration of total enteral feeding (exclusive and/or partial) was 207 days [120–555] and of exclusive enteral feeding, 45 days [18–80].

According to PRS status, 19/24 patients (79%) with iPRS and 22/26 (85%) with sPRS needed enteral feeding. Very severe feeding disorders (duration of enteral feeding >12 months) were observed in 24 % of the patients (Table 4), and were more frequent in patients with sPRS (*p* < 10^−2^). Using univariate analysis, differences were observed between very severe feeding disorders and the other groups. No differences were observed between the other groups. Duration of enteral feeding was significantly longer in patients with severe respiratory disorders (*p* = 0.01). Duration of enteral feeding was not significantly longer in patients with severe retrognathia and glossoptosis.

**Table 4:** Longer durations of enteral feeding are observed in syndromic than in isolated Pierre Robin sequence.

**Feeding disorders**	**Total (*n* = 50; %)**	**iPRS (*n* = 24; % of iPRS)**	**sPRS (*n* = 26; % of sPRS)**
Presence	41 (82%)	19 (79%)	22 (85%)
Very mild (EF < 3 weeks)	5 (10%)	3 (12.5%)	2 (7.5%)
Mild (EF 3 weeks−3 months)	4 (8%)	2 (8%)	2 (7.5%)
Moderate (EF 3–6 months)	6 (12%)	4 (16.5%)	2 (7.5%)
Severe (EF 6–12 months)	14 (28%)	9 (37.5%)	5 (19%)
Very severe (EF > 12 months)	12 (24%)	1 (4%)	11 (42%)^*^

*iPRS, isolated Pierre Robin sequence; sPRS, syndromic Pierre Robin sequence; EF, enteral feeding*.

* *p < 10^−3^ by Fisher test*.

Using univariate analysis, neurological impairments and laryngomalacia were associated with very severe feeding disorders (>12 months) (OR 18.6, 95% CI 3.9–89.2 and OR 20.4, 95% CI 3,4–122.8; *p* < 10^−2^) and with the need for gastrostomy (*p* = 0.04 and *p* = 0.01).

Using multivariate analysis, severe retrognathia and severe glossoptosis, laryngomalacia, and sPRS status were not predictive factors for long-lasting enteral feeding. Only neurological impairments remained significant (aOR 21.72, 95% CI 3.4–138.63; *p* < 10^−2^) in our model adjusted on the confounding factors (year of birth, prematurity and gender). To note, these three confounding factors were not significant in this model.

## Discussion

We retrospectively studied a series of 50 infants with PRS who were managed homogeneously in a single center, and found no significant association between the grades of anatomic variables and the severity of respiratory and feeding disorders.

The general characteristics of our population are in line with previously reported series. Prematurity showed a rate (8%) agreeing with that in the general population (from 6 to 11%) (13), and was not associated with increased rates of severe respiratory or feeding disorders. Our series includes an equal number of iPRS and sPRS, while most published series reported high rates (up to 61.5%) of non-isolated PRS, under the names syndromic, associated, or PRS-plus (3–6, 12, 14–19). The ratio isolated/non-isolated PRS depends on the origin of the published series: more isolated PRS have been reported by medical teams from primary or secondary centers and by surgical teams; more non-isolated PRS have been reported from tertiary centers and by clinical genetics teams. In addition, diagnostic criteria vary among teams. Some consider that cleft palate belongs to PRS (3–5, 16–18, 20–23), whereas other authors define PRS as the association of retrognathia, glossoptosis, and airway obstruction, with or without cleft palate (12, 14, 15, 19, 24). In our series, syndromic PRS status was found to be a significant predictive factor in our model involving respiratory disorders as dependent variable. This underlines that defining PRS status is crucial, thanks to accurate examination in the neonatal period and close scrutiny during follow-up. Depending on whether they have cleft palate, patients with PRS may have different outcomes, as suggested by Vipulananthan et al. (12) who reported that patients with PRS including cleft palate presented more frequently with early respiratory failure than patients without cleft palate. Because we only included infants with retrognathia, glossoptosis, airway obstruction, and cleft palate, our results cannot be extrapolated to patients with PRS without cleft palate.

Airway obstruction management widely vary among teams dealing with PRS (9). TLA and MDO were not performed in this series because since the end of the 90's we started to use CPAP at home in infants with stridor, airway obstruction, and PRS (25). Our team reported good results and no adverse effect of non-invasive CPAP in these populations, through close monitoring during sleep at home (25–27). Because respiratory disorders may have multifactorial origins in patients with PRS, TLA and MDO may not be efficient (28, 29), particularly in patients with several levels of airway obstruction (30). In addition, these surgical methods may be considered as invasive and excessive to treat a condition that may improve shortly. Patients with iPRS are known to have mostly favorable morphological and functional outcomes (5, 31–34). However, we do not exclude the use of MDO in selected patients with severe airway obstruction mainly due to retrognathia, in particular patients with sPRS and mandibular dysostosis. Of note, the rate of tracheostomy in this series (10%) was similar to those of series using TLA or MDO (9). In our population, laryngoscopic assessment and ventilation monitoring helped deciding the best treatment of upper airway obstruction. In clinical practice, we do recommend systematic polygraphic assessment for all patients with PRS, even if they show no apparent ventilation obstruction.

Feeding difficulties in PRS may be due to multiple factors including sucking–swallowing incoordination, esophageal dysmotility, respiration discomfort, central nervous system disorders, and psychologic factors (10, 24, 35–37). Abnormal development of muscles of the tongue has been reported recently (38). Neonatal feeding difficulties imply nutritional support in order to prevent growth failure. In our series, enteral feeding was required for most patients. Previous series reported high rates of NGT feeding (23–70%) and gastrostomy (up to 42%) (5, 16, 21–23, 39). The authors reported various duration of NGT feeding, from 3 months (23), to more than 18 months (39). In line with Smith and Senders (39) and Meyer et al. (21) we found that enteral feeding was more frequently and more lastingly required in patients with sPRS than in those with iPRS.

Associated features may also influence the severity of feeding and respiratory disorders, foremost among them neurological impairments (40). Herein, neurological impairments were associated with severe respiratory and feeding disorders, knowing that our statistical results may be biased by the sample size and might be confirmed in larger populations.

To conclude, the grades of retrognathia and glossoptosis did not predict the severity of respiratory and feeding disorders in a large series of infants with PRS with cleft palate. From a clinical viewpoint, our study underlines the need for systematic clinical investigations to detect any associated disorders, above all laryngeal abnormalities and neulogical impairement.

## Author contributions

AM and VS contributed to the conception, design, acquisition, analysis and interpretation of data, and the writing of the manuscript. DM, FR, BF, SM, and NL contributed to the management of the patients and to the data collection. VS, NK, M-PV, and AP contributed to the management of the patients. VA contributed to the analysis, interpretation of the data and writing of the manuscript. All authors contributed to the revision of the manuscript and approved the final version.

### Conflict of interest statement

The authors declare that the research was conducted in the absence of any commercial or financial relationships that could be construed as a potential conflict of interest.

## Abbreviations

AHI: Apnea Hypopnea Index
CPAP: continuous positive airway pressure
iPRS: isolated Pierre Robin sequence
MDO: mandibular distraction osteogenesis
NGT: nasogastric tube
sPRS: syndromic Pierre Robin sequence
TLA: tongue lip adhesion.
